# Predicting Career Decision-Making Difficulties: The Role of Trait Emotional Intelligence, Positive and Negative Emotions

**DOI:** 10.3389/fpsyg.2018.01107

**Published:** 2018-07-10

**Authors:** Forouzan Farnia, Fredrick M. Nafukho, K. V. Petrides

**Affiliations:** ^1^Educational Administration and Human Resource Development, Texas A&M University, College Station, TX, United States; ^2^Educational Psychology, University College London, London, United Kingdom

**Keywords:** tait emotional intelligence (Trait EI), career decision-making difficulties, career indecisiveness, positive and negative emotions, trait emotional intelligence questionnaire (TEIQue)

## Abstract

The current study evaluated the relationship between positive and negative emotions, trait emotional intelligence (trait EI) and difficulties in career decision-making. We examined whether trait EI could predict career indecisiveness, a type of career decision-making difficulty, over and above the “Big Five” personality traits. We also examined the mechanism through which trait EI affected career indecisiveness by investigating the mediating role of positive and negative emotions. Survey data were collected from a sample of 600 undergraduate students in a university in southwest United States, who completed questionnaires measuring trait EI, positive emotions, negative emotions, personality traits, and career indecisiveness. Hierarchical multiple regression analyses indicated that trait EI accounted for a significant proportion of the variance in career indecisiveness that was not explained by the “Big Five” personality traits. The parallel mediation analysis revealed that positive and negative emotions partially mediated the relationship between trait EI and career indecisiveness. Using the framework of Fredrickson’s (2001) broaden and build theory of positive emotions, the study provides empirical evidence explaining the mechanism through which trait EI helps individuals cope better with challenging situations in life. Trait EI aids the elicitation of positive emotions and the down-regulation of negative emotions, which, in turn, broaden the range of thoughts and actions that come to mind, helping individuals function more effectively in the context of career decision-making. Implications for career counseling and opportunities for further research are discussed.

## Introduction

Selecting a career can be one of life’s most challenging decisions. People often feel overwhelmed by the amount of information they need to absorb when considering the numerous career paths they could potentially follow (Gati and Levin, 2014). A well-researched topic in career psychology, career decision-making difficulties are defined as “the difficulties encountered by individuals while making career-related decisions. They refer to all problems and challenges that need to be addressed prior to, during, or after the decision-making process" (Saka et al., 2008, p. 403).

Facing the challenge of career decision-making could happen at diverse stages of life. It is observed among high school students, who try to decide on their future career by choosing a major, as well as among college students who might continue to struggle with career decisions even after starting an undergraduate program. As Lichtenstein et al. (2009) further explained:

Throughout the undergraduate years, students… often contemplate professional options with no direct relationship to their undergraduate major. For example, a student with a pre-med degree might choose a graduate program in law while a student with an engineering degree might choose a job in investment banking. Students can wrestle with job and career decisions late into their senior year—and beyond (p. 228).

Broadly speaking, career decision-making difficulties are categorized into two types: Career indecision versus career indecisiveness. Career indecision is a state that most people in modern societies go through at some point in their lives when deciding on their future career. In most cases, it will ultimately be resolved over time, either by individuals themselves or with the help of career counselors (Gati et al., 2011). In contrast, some individuals experience a more chronic type of career decisional difficulties stemming from emotional problems or personality-related factors, called career indecisiveness (Gati et al., 2011). Indecisive individuals are “unable to make a vocational choice no matter how carefully they are led through a decision-making process” (Salomone, 1982, p. 498). The prominent factors associated with career indecisiveness identified in previous research were incorporated by Saka et al. (2008) into a theoretical framework referred to as Emotional and Personality-related Career Decision-Making Difficulties (EPCD). Based on the taxonomy of EPCD, difficulties pertaining to the three clusters of ‘pessimistic views,’ ‘anxiety,’ and ‘self-concept and identity’ lead to career indecisiveness in individuals (Saka et al., 2008).

Career decision-making is a rational process, which involves emotions. Emotional information is critical to “shape the individuals’ judgments, decisions, priorities and actions” (Salovey et al., 2000, p. 506). Rational decision-making strategies are often insufficient unless individuals can emotionally manage the uncertainty, ambiguity, and unpredictability that are involved in the decision-making process (Gelatt, 1989). According to Fredrickson’s (2001) broaden and build theory, positive emotions expand the cognitive context by broadening the range of thoughts and actions that come to mind. Positive emotions result into patterns of thought that are more “flexible (Isen and Daubman, 1984), creative (Isen et al., 1987), integrative (Isen et al., 1991), open to information (Estrada et al., 1997), and efficient (Isen and Means, 1983; Isen et al., 1991)” (Fredrickson, 2001, p. 221). These patterns are useful in the decision-making process, where flexibility towards options, coping with stress, adaptability, and engagement are important keys to success.

Trait EI, which is defined as a constellation of emotional perceptions assessed through questionnaires and rating scales (Petrides et al., 2007), is likely to facilitate the career decision-making process by fostering positive emotions and helping individuals regulate the flow of negative emotions involved in the challenges associated with the transition from school to the world of work. A significant body of evidence has linked EI to fewer career decision-making difficulties (e.g., Di Fabio and Palazzeschi, 2009; Di Fabio et al., 2012, 2013; Di Fabio and Saklofske, 2014; Petrides et al., 2016). However, there is a dearth of information on the role of positive and negative emotions in conjunction with trait EI in predicting career indecisiveness.

Personality traits are significantly related to trait EI (Vernon et al., 2008) as well as career decision-making difficulties (Martincin and Stead, 2015). A specific line of research has been investigating the incremental validity of trait EI over and above the “Big Five” personality dimensions and related constructs (e.g., Andrei et al., 2016; Siegling et al., 2017). Examining the incremental validity of trait EI rules out the rival hypothesis that the variation in the criterion variable is accounted for by personality traits, rather than trait EI.

### Study Aims and Hypotheses

**FIGURE 1:**
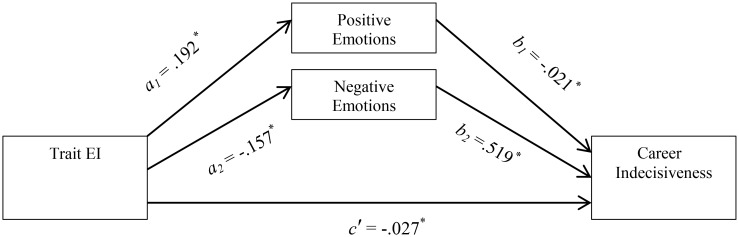
Parallel mediation model for the relationship between trait EI and career indecisiveness via positive and negative emotions. ^∗^*p* < 0.01.

The primary aim of this study was to determine if trait EI can predict career indecisiveness beyond personality traits. A second aim was to examine whether positive and negative emotions mediate the relationship between trait EI and career indecisiveness. Considering the capacity of positive emotions to facilitate the decision-making process by expanding the cognitive context and the array of thoughts and actions that come to mind, and the reverse effect of negative emotions, which limit thought-action repertoires (Tugade and Fredrickson, 2001), it is plausible that trait EI may influence career indecisiveness through the development of strategies to draw on positive emotions and regulate the flow of negative emotions (see **Figure 1**). Accordingly, the following hypotheses were formulated:

*Hypothesis 1:* Trait EI will predict lower levels of career indecisiveness beyond the “Big Five” personality dimensions.
*Hypothesis 2:* Positive and negative emotions will mediate the relationship between trait EI and career indecisiveness.

This study extends the previous work on EI and career decision-making difficulties in several important ways. First, most of the previous studies that examined the associations between EI and career decisional difficulties have focused on career indecision, rather than career indecisiveness. To our knowledge, there is only one previous study (Di Fabio and Saklofske, 2014) on the link between EI and career indecisiveness. Second, the present study assesses the incremental validity of trait EI in predicting career indecisiveness beyond the “Big Five” personality traits. Unless personality traits are controlled for, significant proportions of variance in career decisional difficulties cannot be attributed to trait EI. Last, the study examines the mechanism through which trait EI influences career indecisiveness via positive emotions and negative emotions using a parallel mediation model. Investigating the role of positive and negative emotions, in relation to EI, provides greater insight into explaining how emotionally intelligent individuals are able to successfully cope with stressful encounters (Tugade and Fredrickson, 2001).

## Materials and Methods

### Participants

Undergraduate students at a major research university in the southwestern United States were invited to participate in this study. A total of 739 individuals took part in a comprehensive online survey, which included four separate questionnaires. The responses of those who either left the survey incomplete or were inattentive when answering the questions were removed (139 entries), leaving 600 complete observations. In order to detect inattentive responses, items such as “If you are a human being, please select 4” were inserted among the survey questions. Wrong answers to these questions in addition to repeated selection of the same choice number for several questions in a row were interpreted as signs of inattentive responding.

The minimum required sample size for a multiple regression analysis was determined using the XLSTAT statistical package. According to the results, at least 500 observations were needed in order to work with the recommended power of 0.8 to detect an effect size of 0.02. The final sample comprised 46% male and 54% female undergraduate students, mostly between 18 and 21 years of age (93%). Most were college sophomores (38%), juniors (28%), and seniors (33%) with very few freshmen (1.1%). The sample consisted mostly of United States citizens (98%), with 70% classified as white, 19% as Hispanic, 3.3% as African American, 0.5% as Native American, 6% as Asian-American, and 2% as other.

### Measures

The survey incorporated four separate questionnaires, all of which were valid, reliable, and well-documented in terms of psychometric characteristics.

#### Trait Emotional Intelligence Questionnaire-Short Form (TEIQue-SF)

The TEIQue-SF comprises 30 items responded to on a seven-point Likert scale (Petrides, 2009). Cronbach’s alpha reliability for the global score on the present sample was 0.88.

#### Emotional and Personality Career Decision-Making Difficulties Scale-Short Form (EPCD-SF)

EPCD-SF is a 25- item self-report questionnaire measuring career indecisiveness. Items are responded to on a nine-point Likert scale (Saka et al., 2008). Cronbach’s alpha reliability on the present sample was 0.92.

#### Big Five Inventory (BFI)

The BFI is a 44-item self-report questionnaire based on a five-point Likert scale measuring the personality traits of Extraversion, Agreeableness, Conscientiousness, Neuroticism, and Openness to Experience (John et al., 2008). Cronbach’s alpha reliabilities on the present sample were 0.88 for Extraversion, 0.79 for Agreeableness, 0.80 for Conscientiousness, 0.81 for Neuroticism, and 0.79 for Openness.

#### Positive and Negative Affect Scale (PANAS)

The PANAS was included in the survey to measure positive and negative emotions. It consists of two scales, Positive Activation (PA) and Negative Activation (NA). Each scale contains 10 items, responded to on a five-point Likert scale (Watson et al., 1988). Cronbach’s alpha reliability on the present sample was 0.87 for PA and 0.82 for NA.

### Procedure and Data Analysis

After obtaining IRB approval and permission from the developers to use the four measurement instruments, the researchers sent an invitation e-mail containing the survey link to several professors who taught undergraduate courses at a major research university in southwest United States. Once the data were collected and cleaned, descriptive statistics, Pearson’s correlations, hierarchical regressions, and mediation analysis were conducted to test the study’s hypotheses.

## Results

Means, standard deviationns, and correlation coefficients for all variables in the study are reported in **Table 1**. Variance inflation factors (VIFs) for each independent variable were computed to evaluate the impact of multicollinearity in the data. The VIFs for this sample were within the acceptable interval—the largest being 2.45, indicating that multicollinearity did not threaten the validity of the results. The bivariate correlation matrix of the interval variables in the study revealed significant correlations in the expected direction.

**Table 1:** Means, standard deviations, and correlations for trait EI, the “Big Five” personality traits, positive emotions, negative emotions and career indecisiveness.

Variables	*M*	*SD*	1	2	3	4	5	6	7	8	9
(1) Trait EI	155.25	19.38	1								
(2) Career indecisiveness	4.07	1.36	−0.570*	1							
(3) Positive emotions	35.91	6.80	0.548*	−0.369*	1						
(4) Negative emotions	21.36	6.55	−0.466*	0.455*	−0.211*	1					
(5) Extraversion	3.43	0.84	0.426*	−0.291*	0.411*	−0.127*	1				
(6) Agreeableness	3.98	0.59	0.339*	−0.162*	0.230*	−0.300*	0.142*	1			
(7) Conscientiousness	3.75	0.61	0.425*	−0.279*	0.299*	−0.274*	0.063	0.308*	1		
(8) Neuroticism	2.68	0.74	−0.599*	0.508*	−0.423*	0.571*	−0.225*	−0.268*	−0.221*	1	
(9) Openness	3.41	0.64	0.262*	−0.083	0.253**	−0.024	0.154*	0.134*	0.093	−0.137*	1

*n = 600, ^∗^ p < 0.01.*

To test Hypothesis 1, a hierarchical multiple regression analysis was conducted. In the first step, the control variables, including the “Big Five” personality traits (Agreeableness, Openness to Experience, Extraversion, Conscientiousness, and Neuroticism) were entered. Trait EI was entered in the second step to obtain its added value in explaining variance in career indecisiveness.

The “Big Five” personality traits together accounted for 32% of the variance in career indecisiveness, *R*^2^ = 0.32, *F*(5,594) = 55.45, *p* < 0.001. When trait EI was added at the second step, the regression model remained significant, *F*(6,593) = 59.78, *p* < 0.001, with trait EI accounting for an additional 6% of variance in career indecisiveness. The regression coefficient for trait EI (−0.025) was significantly negative *t*(593) = 7.47, *p* < 0.001, indicating that it was associated with lower levels of career indecisiveness, holding constant the control variables in the model (see **Table 2**). The results showed that high trait EI students tended to experience less career indecisiveness than their low trait EI peers, after controlling for their standing on the “Big Five” personality dimensions.

**Table 2:** Summary of the hierarchical regression analysis with career indecisiveness as the criterion variable.

Variable	*R*^2^	Δ*R*^2^	*b*	β	SE B	*F*	Δ*F*	95% CI
**Step 1**	0.318*					55.45*		
Extraversion			−0.308*	−0.192*	0.056			[−0.418, −0.198]
Agreeableness			0.053	0.023	0.085			[−0.113, 0.219]
Conscientiousness			−0.393*	−0.178*	0.080			[−0.549, −0.236]
Neuroticism			0.789*	0.432*	0.066			[0.659, 0.920]
Openness			0.043	0.02	0.072			[−0.099, 0.185]
**Step 2**	0.377*	0.059*				59.78*	55.837*	
Extraversion			−0.143	−0.089	0.058			[−0.257, −0.028]
Agreeableness			0.117	0.051	0.081			[−0.043, 0.277]
Conscientiousness			−0.171	−0.077	0.082			[−0.332, −0.010]
Neuroticism			0.498*	0.273*	0.075			[0.351, 0.644]
Openness			0.136	0.065	0.070			[−0.002, 0.274]
Trait EI			−0.025*	−0.365*	0.003			[−0.032, −0.019]

*n = 600, ^∗^p < 0.01, CI = Confidence Interval.*

To test Hypothesis 2, we employed an SPSS macro, PROCESS (Model 4; Hayes, 2013), to estimate the indirect effect of trait EI on career indecisiveness via the two mediators (positive emotions and negative emotions). As illustrated in **Figure 1**, those higher in trait EI reported a higher level of positive emotions (Path a_1_ in **Figure 1**, *b* = 0.192, *SE* = 0.012, *p* < 0.001), which, in turn, was linked with lower levels of career indecisiveness (path b_1_ in **Figure 1**, *b* = −0.0216, *SE* = 0.007, *p* = 0.006). In contrast, higher trait EI was linked to less negative emotions (path a_2_ in **Figure 1**, *b* = −0.157, *SE* = 0.012, *p* < 0.01), which, in turn, were associated with lower career indecisiveness (path b_2_ in **Figure 1**, *b* = 0.519, *SE* = 0.007, *p* < 0.001).

To calculate 95% bias-corrected confidence intervals (CI) for the point estimate, 10,000 bootstrap samples were generated. When the CI of the indirect effect of a mediator do not include 0, it is considered statistically significant (Hayes, 2013). As shown in **Table 3**, a 95% bias-corrected confidence interval showed that the indirect effect of trait EI on career indecisiveness via positive emotions (*b* = −0.004) was consistently less than zero (−0.0074 to −0.0011). The indirect effect of trait EI on career indecisiveness via negative emotions (*b* = −0.008) was also consistently less than zero (−0.0111 to −0.0056). The results revealed the existence of a parallel mediation effect, as trait EI was indirectly related to career indecisiveness via its relationship to positive and negative emotions. The mediation was partial, since the unstandardized beta coefficient for the direct effect of trait EI on career indecisiveness (path c′ in **Figure 1**) after controlling for positive and negative emotions was significant (*b* = −0.027, *SE* = 0.003, *p* < 0.001).

**Table 3:** Summary results of mediation analyses.

	Coefficients	SE	BC Bootstrap 95% CI	
			Upper	Lower
Direct effect of trait EI on career indecisiveness	−0.027*	0.003	−0.0337	−0.0218
Indirect effect of trait EI on career indecisiveness via positive emotions	−0.004*	0.0016	−0.0074	−0.0011
Indirect effect of trait EI on career indecisiveness via negative emotions	−0.008*	0.0014	−0.0111	−0.0056

*^∗^p < 0.01.*

## Discussion

The results of the multiple hierarchical regression analysis supported Hypothesis 1, trait EI explained a significant proportion of variance in career indecisiveness that was not explained by the “Big Five” personality dimensions. More specifically, trait EI increased the predictive ability of the regression model by 6%. While this may seem small, in the presence of numerous contextual and situational factors that can affect the career decision-making process for an individual, it represents a very considerable increase. Demonstrating the role of trait EI in predicting career indecisiveness over a and above standard measurement of personality is particularly meaningful in that it should give pause to those who argue that EI is merely a “repackaging” of personality traits (see also Andrei et al., 2016).

It is likely trait EI helps people decode the emotional weight that they associate with different options, and have more faith in their choices, decreasing the feelings of confusion, hesitation, and self-doubt that are often experienced by indecisive individuals. In contrast, low trait EI might eventually result in inability to control and eliminate causes of career indecisiveness, such as pessimistic views towards the process of career decision-making and its outcomes or the feeling of guilt, arising from favoring a career choice that is not approved by significant others (Saka et al., 2008).

The study results also demonstrated a partial mediation effect of positive and negative emotions in the relationship between trait EI and career indecisiveness. High trait EI individuals are better able to elicit positive emotions and regulate any negativity that tends to arise in challenging situations, such as career decision-making. The enhanced experience of positive emotions and the down-regulation of negative emotions subsequently create patterns of thoughts that are more flexible, inclusive, receptive, creative, and variable (Tugade and Fredrickson, 2001), thus improving the functionality and career decision-making of individuals.

The study has important implications for career counselors. Paying attention to the emotional and personality-related aspects of career decision-making difficulties can assist in accurately diagnosing the sources of difficulty and developing appropriate interventions. If a client is experiencing significant career indecisiveness, the underlying emotional issues often cannot be resolved through typical consultations limited to supplying career information and rational decision-making strategies (Gati and Levin, 2014). Interventions focusing on clients’ emotions should be incorporated, with one option being trait EI training for the client (e.g., Nelis et al., 2011).

This study focused exclusively on undergraduate students at a major research university in the United States. However, the situation of those college students living in other geographic or cultural regions may not be identical to that of the sample of this study. Furthermore, career indecisiveness could be a concern for a wider variety of individuals, including high school students and professionals who are considering a change of career. The results of this study should not be extrapolated beyond the specific context and circumstances of its research participants. This leaves valuable opportunities to expand the research into other populations and to determine if the trait EI effects on career decision-making difficulties can be further generalized. Future research could also incorporate a greater number of background variables, such as participants’ ethnicity, age, socioeconomic status, and field of study, and look for interaction effects between them and trait EI in the prediction and resolution of various types of career decision-making difficulties.

## Disclosures

This research did not receive any specific grant from funding agencies in the public, commercial, or not-for-profit sectors. The authors have no financial or personal relationships with individuals or organizations that could inappropriately influence the study.

## Ethics Statement

This study was carried out in accordance with the recommendations of Texas A&M University, Institutional Review Board (IRB) for the protection of human research participants. The protocol was approved by the Texas A&M, IRB. All subjects gave written informed consent in accordance with the Declaration of Helsinki.

## Author Contributions

FF conducted the research as part of her Ph.D. degree requirements, wrote and revised the manuscript. FN supervised the research as FF’s dissertation committee chair. KP collaborated with FF in revising the manuscript and generating the final draft.

## Conflict of Interest Statement

The authors declare that the research was conducted in the absence of any commercial or financial relationships that could be construed as a potential conflict of interest.
